# Metagenomic insights into the complex viral composition of the enteric RNA virome in healthy and diarrheic calves from Ethiopia

**DOI:** 10.1186/s12985-025-02821-8

**Published:** 2025-06-07

**Authors:** Julia Bergholm, Tesfaye Sisay Tessema, Anne-Lie Blomström, Mikael Berg

**Affiliations:** 1 Department of Animal Biosciences, Swedish University of Agricultural Sciences, Uppsala, Sweden; 2 Institute of Biotechnology, Addis Ababa University, Addis Ababa, Ethiopia

**Keywords:** Calf diarrhea, Viral metagenomics, Ethiopia, Africa, High-throughput sequencing

## Abstract

**Background:**

Viruses and the virome have received increased attention in the context of calf diarrhea and with the advancement of high-throughput sequencing the detection and discovery of viruses has been improved. Calf diarrhea, being the main contributor to calf morbidity and mortality, is a major issue within the livestock sector in Ethiopia. However, studies on viruses and the virome in calves is lacking in the country. Therefore, we utilized viral metagenomics to investigate the diversity of RNA viruses in healthy and diarrheic calves from central Ethiopia.

**Methods:**

Fecal material from 47 calves were collected, pooled, and sequenced using Illumina. Following sequencing, the virome composition and individual viral sequences were investigated using bioinformatic analysis.

**Results:**

The metagenomic analysis revealed the presence of several RNA viruses, including rotavirus and bovine coronavirus, known causative agents in calf diarrhea. In addition, several enteric RNA viruses that have not been detected in cattle in Ethiopia previously, such as norovirus, nebovirus, astrovirus, torovirus, kobuvirus, enterovirus, boosepivirus and hunnivirus were identified. Furthermore, a highly divergent viral sequence, which we gave the working name suluvirus, was found. Suluvirus showed a similar genome structure to viruses within the *Picornaviridae* family and phylogenetic analysis showed that it clusters with crohiviruses. However, due to its very divergent amino acid sequence, we propose that suluvirus represent either a new genus within the *Picornaviridae* or a new species within crohiviruses.

**Conclusions:**

To our knowledge, this is the first characterization of the RNA virome in Ethiopian cattle and the study revealed multiple RNA viruses circulating in both diarrheic and healthy calves, as well as a putative novel virus, suluvirus. Our study highlights that viral metagenomics is a powerful tool in understanding the divergence of viruses and their possible association to calf diarrhea, enabling characterization of known viruses as well as discovery of novel viruses.

**Supplementary Information:**

The online version contains supplementary material available at 10.1186/s12985-025-02821-8.

## Background

Diarrheic disease is one of the main causes of mortality and morbidity in neonatal calves and it is a complex disease that is caused by infectious, environmental, and host factors. Bacteria, protozoans, and viruses can be causative agents in calf diarrhea and co-infection with numerous pathogens is commonly observed [1]. Over the years, viruses and the virome composition have received increased attention in the context of calf diarrhea, and various RNA viruses have been associated with the disease [2]. Rotavirus A (RVA) and bovine coronavirus (BCoV) are two known and common viral causes of neonatal calf diarrhea. RVA is a group of segmented double-stranded RNA viruses that can infect a wide range of hosts, including humans. In calves, they are considered a major enteric pathogen and often cause diarrheic disease in calves below two weeks of age [1, 3]. BCoV is a single-stranded RNA virus associated with both gastrointestinal and respiratory disease in cattle [4]. Other RNA viruses that have been linked to neonatal calf diarrhea include astrovirus, enterovirus, kobuvirus, nebovirus, norovirus and torovirus [2]. However, these viruses have been identified in both diarrheic and non-diarrheic calves and their role in clinical disease, if any, is not fully determined [2].

With the advancement of high-throughput sequencing (HTS) the detection, discovery, and surveillance of viruses in calf diarrhea and other livestock diseases has been greatly improved [5, 6], and both previously known and novel viruses have been characterized in cattle in recent years [7–9]. Furthermore, viral metagenomics allows for the characterization of all viruses in a sample simultaneously, which is a vital step in understanding calf diarrhea, where co-infections play an important role.

In Ethiopia, the cattle industry is heavily impacted by diarrheic disease, and it is the main contributor to mortality and morbidity in neonatal calves [10–12]. At the same time, studies on the infectious agents in calves, primarily viruses, are lacking in the country. Furthermore, to our knowledge, there has been no study in Africa utilizing viral metagenomics in the context of calf diarrhea to this date. In a previous study on calves from the central regions of Ethiopia, we detected the enteric pathogens *Cryptosporidium spp., E. coli* K99 +, RVA, and BCoV using the conventional screening methods antigen-ELISA and qPCR. However, several samples from diarrheic calves in that study tested negative for all four pathogens [13]. Therefore, we decided to apply viral metagenomics to investigate the RNA virome composition in both the diarrheic and non-diarrheic calves, to find out if other viral pathogens exist in association to calf diarrhea.

## Methods

### Study area, sample preparation and sequencing

Sample collection and preparation was performed in January and February 2023 as previously described [13]. In summary, 47 fecal samples were collected from diarrheic and non-diarrheic calves (< 2 months of age) from farms in the towns Sebeta, Holeta, Sululta, and Bishoftu in central Ethiopia. The fecal samples were homogenized in DNA/RNA Shield™ (10% w/v) and homogenized using ZR BashingBead™ Lysis Tubes (Zymo research, Irvine, CA, USA) and a Vortex-Genie in combination with a horizontal tube holder (Thermo Fisher Scientific, Waltham, MA, USA). The homogenates were centrifuged at 12,000 × *g* for 1 min and the supernatant was collected and filtered using 0.45 uM centrifugal membrane filters (Ultrafree®-MC centrifugal Filter, Darmstadt, Germany). Following filtering, RNA was extracted using a combination of TRIzol (Invitrogen, Carlsbad, USA) and GeneJet RNA extraction columns (Thermo Fisher Scientific, Waltham, USA). In preparation of sequencing, the RNA from all samples was pooled according to sample location and health status of the calves (diarrheic and non-diarrheic), resulting in a total of ten pools. All pools were then subjected to DNase treatment and concentration using a combination of the RNase-Free DNase set and RNAeasy mini elute kit (Qiagen, Hilden Germany). Next, ribosomal RNA was removed using Ribo-Zero Plus rRNA depletion kit (Illumina, San Diego, USA), followed by random amplification using the Ovation® RNA-Seq System V2 (Tecan, Männedorf, Switzerland) according to the manufacturer’s instructions. The amplified RNA pools were sent to the SNP&SEQ Technology Platform in Uppsala, Sweden for library preparation and sequencing using the SMARTer ThruPLEX DNA-seq kit (Takara Bio, San Jose, USA) and the NovaSeq 6000 sequencing system (Illumina, San Diego, USA).

### Bioinformatic analysis

The sequencing data was analyzed using a set of different bioinformatic tools. First, the raw reads were trimmed, de-duplicated, and quality checked using fastp (v0.23.4) [14]. Trimmed reads were then taxonomically classified on viral level using Kraken2 (v2.0.8-beta) [15] and the Kraken2 viral database (updated 2024/12/28). The relative abundance of viral families in each pool was estimated by Bracken (v2.5) [16]. The relative abundance was visualized with BrackenPlot [17], showing the ten most abundant viral families by mean. In parallel to taxonomic classification with Kraken2, the trimmed reads were also subjected to de-novo assembly using MEGAHIT (v1.2.9) [18]. The de-novo assembled contigs were taxonomically classified by BLASTx using DIAMOND (v2.1.6.160) [19] and imported into MEGAN6 (v6.24.4) [20] for visualization. In parallel, the contigs were also classified using Kraken2 and visualized using Pavian [21]. Longer viral contigs of interest were extracted and imported into Geneious Prime (v2024.0.5) for annotation of ORFs and manual curation. This was followed by confirmation of the classification in DIAMOND by BLASTn, BLASTp, and BLASTx. Viral sequences that were obtained from contigs containing the complete coding part of the viral genome were further explored with phylogenetic analysis. Sequences were aligned with MAFFT [22] and imported into IQ-TREE (v.1.6.12) [23] where maximum likelihood trees were constructed using 1,000 ultrafast bootstrap replicates according to the best substitution model by lowest BIC score. The phylogenetic trees were visualized in Interactive Tree of Life (iTOL) v7.0 [24]. All the viral genome sequences and their corresponding accession numbers are listed in Supplementary Table 1.

### Genetic characterization of suluvirus

To genetically characterize suluvirus, additional steps of analysis were performed. First, the trimmed reads were mapped back to the contig using Bowtie2 to estimate the coverage of the viral sequence. Secondly, the protein-encoding region of the viral sequence was analyzed with EMBL-EBI’s protein sequence analysis and classification tool InterProScan [25]. Phylogenetic analysis of the 3D and P1 aa sequences with genomes of genera within the *Paaviviridae* subfamily (Family: *Picornaviridae*) was performed as in the previous section. Lastly, two PCRs were designed to verify the viral sequence. First, a PCR targeting a 382 bp region of the contig was designed to screen samples (from pool J08) for suluvirus (Sulu-S-FW: 5´- CCCATCATGCAAATCCGCTG-3´, Sulu-S-RV: 5’- ACCAGTAACTGCTGATGGGC—3’). A second PCR was designed to further characterize any individual suluvirus-positive samples, by amplifying a longer section (924 bp) of the suluvirus genome (Sulu-L-FW: 5´- AATTGCCAAACAGCAGCAGG—3’, Sulu-L-RV: AACATCAGCAGCATGTCCCA—3’). Both PCR reactions were run for 35 cycles in a 25 µL reaction with Invitrogen™ Platinum Superfi DNA Polymerase (Invitrogen, Carlsbad, CA, USA) according to manufacturer’s instructions. PCR products were visualized on a 1% agarose gel and purified using the GeneJET™ gel extraction kit (Thermo Fisher Scientific, Waltham, MA, USA). Purified products were subsequently sent for sequencing at Macrogen Europe.

### qPCR screening of individual viruses

All 47 individual samples were screened for BNoV and suluvirus using qPCR. Primers and probes used in the viral screening are provided in Supplementary Table 2. cDNA was synthesized from the original RNA extracts using the SuperScript III reverse transcriptase (Invitrogen, Carlsbad, CA, USA) according to the manufacturer’s instructions. For qPCR detection, 2 µL of cDNA was used as template in a 20 µL reaction using the iTaq Universal Probes Supermix or the iTaq universal SYBR® Green kit (Bio-Rad, Hercules, CA, USA) according to the manufacturer’s instructions. For BNoV 600 nM of primers and 150 nM of probe was used and the reaction was run with the following cycling conditions: 95 °C for 30 s followed by 45 cycles of 95 °C for 15 s and 60 °C for 60 s. For suluvirus, 500 nM of primers were used in the reaction and the qPCR reaction was as follows: 95 °C for 2 min followed by 40 cycles of 95 °C for 10 s and 60 °C for 30 s; and a melt curve step 65 °C to 95 °C (increment 0.5 °C 5 s).

## Results

### Sequencing output and virome characterization

**Fig. 1:**
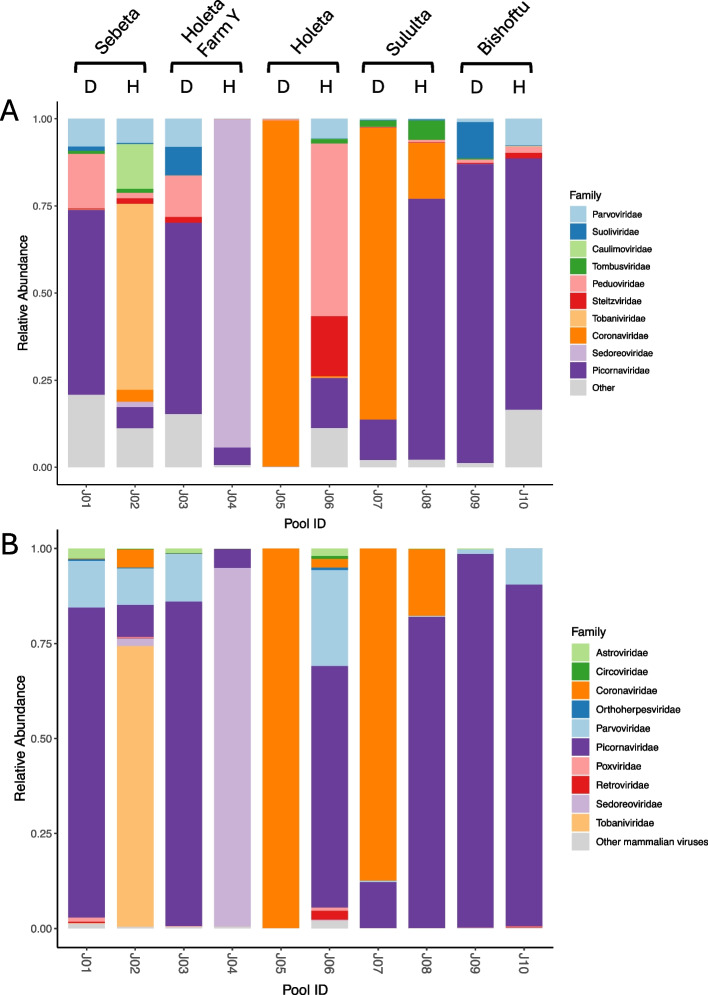
Bracken plot showing the relative abundance of viral reads in each pool. Sample location and health status (D: Diarrheic, H: Non-diarrheic) is displayed for all pools. **A** Displays the top ten most abundant viral families by mean. Remaining viral families are grouped as “Other”. **B** Displays the top ten most abundant mammalian viral families by mean. Remaining viral families are grouped as “Other Mammalian Viruses”

A total of 47 calves were sampled and divided into ten pools depending on health status and sample location (Table 1). For each pool between 18 and 35 million paired reads were obtained post trimming. Of the trimmed reads, 0.07–21.83% were classified as viral when analyzed with Kraken2 (Table 1). The relative abundance of the viral reads on family level was estimated using Bracken and the ten most abundant viral families by mean are visualized in Fig. 1A. Remaining reads classified in other viral families were grouped in one category as “Other”. The ten most abundant viral families across the pools were the RNA viruses *Tombusviridae, Steitzviridae, Tobaniviridae, Coronaviridae, Sedoreoviridae* and *Picornaviridae,* and the DNA viruses *Parvoviridae, Suoliviridae, Caulimoviridae,* and *Peduoviridae* (Fig. 1A).

**Table 1:** Description and sequencing output from each pool

Pool ID	Health status	Sample location	No. of samples	Total raw reads	Paired trimmed reads	Viral reads (% of trimmed reads)
J01	Diarrheic	Sebeta	7	56,189,418	18,044,061	23,257 (0.13)
J02	Non-diarrheic	Sebeta	5	67,451,080	24,041,790	33,792 (0.14)
J03	Diarrheic	Holeta Farm Y	6	115,702,274	29,8177,98	55,184 (0.19)
J04	Non-diarrheic	Holeta Farm Y	6	87,241,456	24,131,705	559,678 (2.32)
J05	Diarrheic	Holeta	5	72,028,634	24,341,612	5,314,023 (21.83)
J06	Non-diarrheic	Holeta	4	71,131,694	23,211,407	17,023 (0.07)
J07	Diarrheic	Sululta	6	62,004,264	24,227,744	213,458 (0.88)
J08	Non-diarrheic	Sululta	4	64,445,228	23,973,930	148,667 (0.62)
J09	Diarrheic	Bishoftu	2	56,032,138	21,880,136	144,325 (0.66)
J10	Non-diarrheic	Bishoftu	2	93,792,634	35,118,000	38,382 (0.11)

To get an overview of what viral families could potentially be involved in bovine enteric disease, the reads were filtered for viral families known to infect mammals. The ten most abundant mammalian viral families were the RNA viruses *Astroviridae, Coronaviridae, Picornaviridae, Retroviridae, Sedoreoviridae* and *Tobaniviridae.* The most abundant mammalian DNA viruses were *Circoviridae, Orthoherpesviridae*, *Parvoviridae* and *Poxviridae* (Fig. 1B). Of the mammalian viruses, *Picornaviridae* and *Parvoviridae* were the most abundant and could be found in all sequencing pools, both diarrheic and non-diarrheic. Reads classified as *Astroviridae* and *Coronaviridae* were also identified in multiple sequencing pools, but at a lower overall abundance. The families *Sedoreoviridae* and *Tobaniviridae* made up the majority of viral reads in pool J04 and J02, respectively, but were less frequent across other sequencing pools. Lastly, *Retroviridae*, *Circoviridae*, *Orthoherpesviridae*, and *Poxviridae* were less abundant, with fewer than 200 reads reported for each family per pool (data not shown).

Longer viral contigs of RNA viruses that are associated with enteric disease in cattle were further investigated and are described below.

### Investigation of viral contigs

#### Coronaviridae

Contigs of various length assigned to the *Coronaviridae* family were found in five sequencing pools (two diarrheic and three non-diarrheic). Two BCoV sequences containing the complete coding region were identified, one in pool J05 (Diarrheic, Holeta) (BCoV/Cow/ETH/2023/25), and one in pool J07 (Diarrheic, Sululta) (BCoV/Cow/ETH/2023/40). Alignment of the two full-length sequences showed a 97.2% sequence identity. When aligning the spike gene, a lower nucleotide identity between the two strains was observed, with 91.1% of the nucleotides being identical. BLASTn search identified a classical BCoV strain from France (MG757138.1) as the closest match to BCoV/Cow/ETH/2023/25, with 99.19% nt identity. For BCoV/Cow/ETH/2023/40, the closest match was a dromedary camel coronavirus (DcCoV) (MN514962.1) isolated from an Ethiopian dromedary camel, with 97.87% nt identity.

**Fig. 2:**
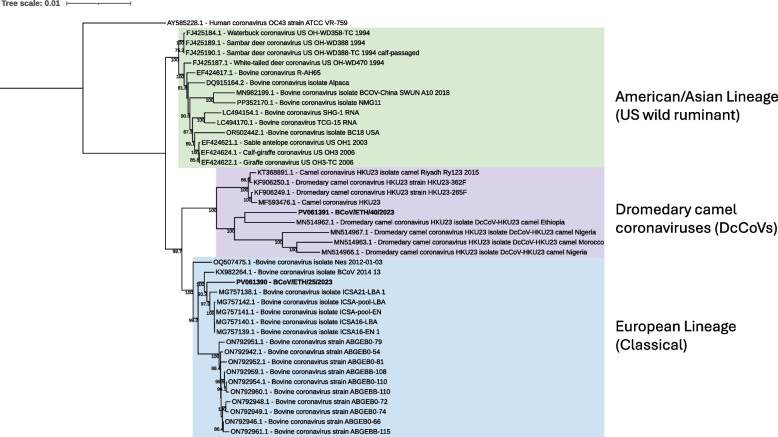
Maximum likelihood tree of full-length genome sequences of BCoV and DcCoV. The tree is out grouped by the human coronavirus OC43 (AY585228.1) with bootstrap values ≥ 70 displayed. Sequences belonging to the American/Asian lineage are highlighted by green, dromedary camels by purple, and the European lineage by blue. The viral sequences identified in the current study are indicated by bold text (PV061390-91)

Phylogenetic analysis of the two isolated sequences together with reference sequences resulted in three distinct groups. The American/Asian lineage (US wild ruminant) with BCoV sequences isolated in the Americas and Asia. The second group included coronaviruses isolated from dromedary camels (DcCoV) in Africa and the Middle East. Lastly, the third group consisted of the European lineage (Classical) with BCoV isolates from Europe. BCoV/Cow/ETH/2023/25 clustered together with the classical BCoV strains in the European lineage while BCoV/Cow/ETH/2023/40 clustered with the isolates from dromedary camels, forming a monophyletic group with the DcCoV isolated from Ethiopia (Fig. 2). The BLAST analysis together with the phylogenetic results demonstrates that two distinct strains were identified, one classical BCoV strain and one BCoV strain more similar to DcCoVs.

#### Astroviridae

**Fig. 3:**
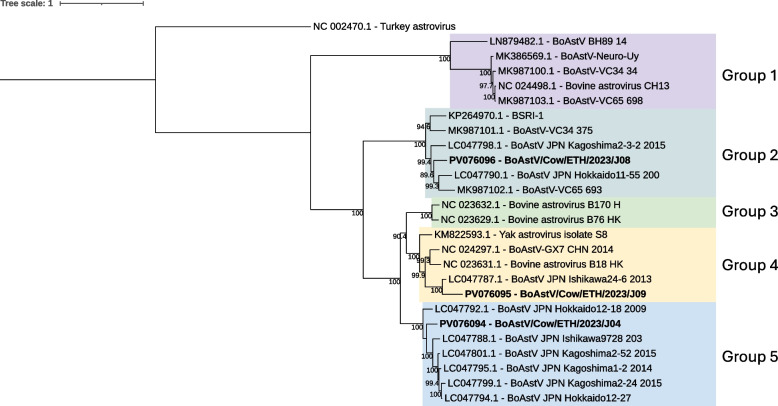
Maximum likelihood tree of the full-length genome sequence of astrovirus. The tree is out grouped by a turkey astrovirus (NC_002470.1) with bootstrap values ≥ 70 displayed. Different groups are highlighted by color, group 1 (purple), group 2 (grey), group 3 (green), group 4 (yellow), and group 5 (blue). The viral sequences obtained in the current study is indicated by bold text (PV076094-96)

Viruses within the *Astroviridae* family have a single-stranded positive-sense RNA genome and contigs classified as astroviruses were found in all sequencing pools except for J10 (Non-diarrheic, Bishoftu). Full-length genomes classified as a bovine astrovirus (BoAstV) could be extracted from pool J04, and J09. Consistent with other astroviruses the genomes contained two open reading frames, ORF1 and ORF2. In addition, a partial BoAstV contig of 2,061 nucleotides was found in pool J08. The highly conserved ORF1a/b ribosomal frameshift site (AAAAAAC) of astroviruses was identified in all three BoAstV sequences. The top matching hit when analyzing BoAstV/Cow/ETH/2023/J04 using BLASTn was a BoAstV isolate from China (ON682283) with 100% query cover and 85.43% nt identity. For BoAstV/Cow/ETH/2023/J08 the top hit was another isolate from China (ON624271) with 100% query cover and 87.05% nt identity. The sequence from pool J09, BoAstV/Cow/ETH/2023/J09, was most similar to an isolate from France (OR261089) with 100% query cover and 82.64% nt identity. BoAstVs can be further divided into five separate groups by phylogenetic analysis of the complete nucleotide sequence [26]. BoAstV/Cow/ETH/2023/J04 grouped together with sequences from group 5, BoAstV/Cow/ETH/2023/J08 with group 2, and BoAstV/Cow/ETH/2023/J09 with group 4 (Fig. 3).

#### Caliciviridae

**Fig. 4:**
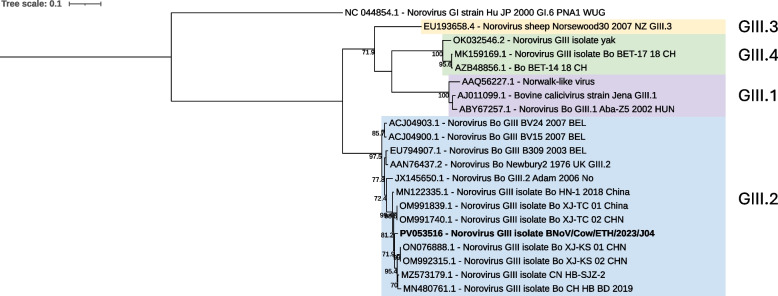
Maximum likelihood tree of the VP1 aa sequence of norovirus. The tree is out grouped by human norovirus G1 strain (NC_044854.1) with bootstrap values ≥ 70 displayed. Different genotypes are highlighted by color, GIII.1 (purple), GIII.2 (blue), GIII.3 (yellow), and GIII.4 (green). The viral sequence identified in the current study is indicated by bold text (PV053516)

Contigs classified into two separate genera within *Caliciviridae*, a positive-sense single-stranded RNA virus family, were identified. In pool J04 (Non-diarrheic, Holeta Farm Y), a full-length bovine norovirus genome (BNoV) (7,299 bp) was found (BNoV/Cow/ETH/2023/J04). ORF analysis of the sequence revealed three ORFs (polyprotein, VP1, and VP2), typical for the norovirus genus, and BLASTx analysis of the three ORFs revealed the genogroup classification GIII. Noroviruses can be further classified into genotypes by analyzing the amino acid sequence of the VP1 protein. Phylogenetic analysis of the VP1 protein sequence together with representative sequences of the four genotypes within genogroup GIII showed that the identified sequence clustered within the GIII.2 genotype clade (Fig. 4). This was further confirmed by the BLASTx analysis, with the top hits for the VP1 sequence all belonging to genotype 2 of the GIII genogroup.

In addition to the full-length BNoV GIII.2 genome, a contig of 1,047 nucleotides belonging to the nebovirus genus (Nebovirus/Cow/ETH/2023/J09) was identified in the diarrheic pool from Bishoftu (J09). BLASTx analysis confirmed that the contig belonged to nebovirus, encoding a partial region of the ORF2 polyprotein.

#### Picornaviridae

**Fig. 5:**
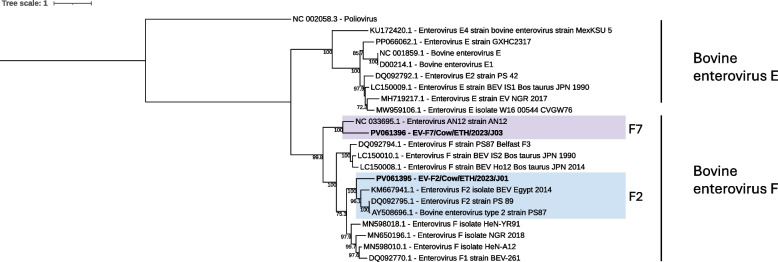
Maximum likelihood tree of the polyprotein aa sequence of bovine enteroviruses. The tree is out grouped by poliovirus (NC_0002058.3) with bootstrap values ≥ 70 displayed. Different genotypes are highlighted by color, F7 (purple) and F2 (blue). The viral sequences identified in the current study is indicated by bold text (PV061395-96)

Both reads and contigs classified as *Picornaviridae,* a family of single-stranded positive-sense RNA viruses, could be found across all sequencing pools. Contigs from four separate viral genera were further investigated; kobuvirus, enterovirus, hunnivirus and boosepivirus. Bovine kobuvirus (BKV) contigs of various length were found in all sequencing pools and the longest, a contig of 4,011 bp, encoding a partial region of the genome could be retrieved from pool J04 (non-diarrheic, Holeta Farm Y). The closest match was a BKV isolated in China (ON730709.1) with 87.28% nt identity when analyzed with BLASTn. Two contigs classified as bovine enterovirus (BEV) could be extracted from two separate pools, J01 (Diarrheic, Sebeta) and J03 (Diarrheic, Holeta Farm Y). The contig from pool J01 (EV-F2/Cow/ETH/2023/J01), was 7,337 nucleotides long, encoding a complete 2,168 aa polyprotein. BLASTp analysis of the polyprotein sequence revealed a BEV isolated from China in 2021 as the top hit (ON986117). The second contig (EV-F7/Cow/ETH/2023/J03) had a 7,356 nucleotides long genome and encoded a full-length polyprotein of 2,175 aa. The closest match on protein level was a BEV from a cow in Japan (NC_033695). Based on phylogenetic analysis of the polyprotein aa sequence, the two BEV sequences were classified as genotype F2 (EV-F2/Cow/ETH/2023/J01) and genotype F7 (EV-F7/Cow/ETH/2023/J03), respectively (Fig. 5).

Hunnivirus (HuV) contigs were also found in almost all sequencing pools, with the exception of pool J06 (Non-diarrheic Holeta) and J10 (Non-diarrheic, Bishoftu). A complete HuV genome sequence could be extracted from pool J07 (Diarrheic, Sululta), and analysis with both BLASTn and BLASTp revealed a bovine HuV isolated in Hungary (NC_018668.1) to be the closest match. The similarity on nt level (complete genome sequence) was 86.22% and on aa level (polyprotein sequence) 94.61%. Lastly, a sequence of 7,520 nucleotides assigned as boosepivirus (BooV) was identified in pool J06 (Non-diarrheic Holeta). BLASTn search of the full-length sequence identified a BooV B strain from China (OP554215.1) as the closest match, with 86.75% nucleotide identity. The sequence encoded a single polyprotein of 2,341 aa, similar to other BooVs, and was most similar to the same BooV B strain from China (OP554215.1) with 97.57% aa identity.

#### Sedoreoviridae

Contigs classified within the rotavirus genus of the *Sedoreoviridae* family were found in several pools. A majority of the identified contigs were not full-length and were identified in pools containing multiple RVA positive samples. However, in pool J02 (Non-diarrheic, Sebeta) RVA contigs belonging to a single positive sample were identified (Supplementary Table 3). Investigation of the RVA contigs from pool J02 in MEGAN6 resulted in the identification of all RVA segments except for NSP1 and NSP5 (Table 2). RVAs are classified according to their full genome layout, Gx-P[x]-Ix-Rx-Cx-Mx-Ax-Nx-Tx-Ex-Hx, representing the genotypes of VP7-VP4-VP6-VP1-VP2-VP3-NSP1-NSP2-NSP3-NSP4-NSP5/6 [27]. Characterization of the segments using BLASTn revealed the RVA genotype of the sample in pool J02 to be G24-P[33]-I2-R2-C2-M2-Ax-N2-T9-E2-Hx (Table 2).

**Table 2:** Table displaying the genotype of each segment in the identified RVA G24P[33] strain, as well as closest match on nucleotide level using BLASTn. Segments that are uncharacterized are indicated by “- “

Segment nr	Segment name	Genotype	BLASTn top hit	nt identity (%)
1	VP1	R2	RVA/Human-wt/IND/RO1-8980/2010/G12P[6] (OR192354.1)	84.15%
2	VP2	C2	RVA/Human-wt/DEU/GER29-14/2014/G6P[9] (KX880440.1)	93.43
3	VP3	M2	RVA/Human-wt/USA/12US1134/2012/G3P[9] (KF500521.1)	92.56
4	VP4	P[33]	Dai-10/G24P[33] (AB573076.1)	94.58
5	NSP1	-	-	-
6	VP6	I2	RVA/Human-wt/JPN/12597/2014/G8P[14] (LC340012.1)	95.89
7	NSP3	T9	Dai-10/G24P[33] (AB573076.1)	93.74
8	NSP2	N2	Rotavirus A Ind/Bo/HR/B85 (JF831950.1)	96.02
9	VP7	G24	Dai-10/G24P[33] (AB573076.1)	88.30
10	NSP4	E2	Bovine rotavirus A strain DK12011 (JN248456.1)	95.87
11	NSP5	-	-	-

#### Tobaniviridae

Three contigs between 6,813–12,189 nt that were classified as torovirus, a single-stranded positive-sense RNA virus within the *Tobaniviridae* family, were identified in the sequencing pool with non-diarrheic calves from Sebeta (J02). The three contigs were subsequently assembled into one full-length contig of 28,386 nt. Mapping of the separate contigs back to the full-length assembled sequence as well as reference BToV Ishikawa/2010 (LC08809) showed that the contigs covered the complete genome. ORF analysis of the assembled contig identified six ORFs (ORF1a/b, S, M, HE and N), typical for toroviruses. BLASTn analysis of the sequence showed the closest match to be a bovine torovirus (BToV) isolated in China (ON337874.1) with 97.87% nt identity.

#### Characterization of a putative novel virus – suluvirus

When exploring pool J08 (Non-diarrheic, Sululta) in MEGAN6, a 7,413 nucleotide long contig classified under the class *Pisoniviricetes* was found. However, the viral sequence displayed low amino acid identity to other viruses within the class. To estimate the read coverage of the contig, all reads from pool J08 were mapped to the contig. The assembly resulted in 16,204 reads being mapped back to the full length contig. To confirm the sequence from the metagenomic assembly, suluvirus-positive samples were analyzed using a PCR targeting a 924 bp region of the contig. One sample generated a product of the expected size and sequencing revealed the 924 bp PCR product to be identical to the sequence of the identified contig, confirming the MEGAHIT-assembled contig (data not shown). We propose to give the viral sequence the preliminary name suluvirus, after the sample location Sululta.

ORF analysis found a single ORF spanning 6,666 nucleotides of the contig. InterProScan analysis of the sequence revealed it to be a polyprotein (2,221 aa), belonging to the helicase/polymerase/peptidase polyprotein, calicivirus-type family (IPR004004). The polyprotein sequence contained matching domains of a picornavirus capsid (IPR001676), a picornavirus/calicivirus coat protein (IPR033703), a helicase superfamily 3 domain (IPR014759), a *Picornavirales* 3 C/3 C-like protease domain (IPR044067), and the catalytic core domain of RdRP belonging to the family *Picornaviridae* (cd23193). BLASTp of the polyprotein sequence revealed suluvirus to have highest similarity with polyprotein sequences from viruses in the order *Picornavirales* and the family *Picornaviridae*. Matching sequences included isolates from a variety of mammals, including bats, rodents, and cattle, with the top hit being the Rousettus bat picornavirus (PP711915) isolated in Kenya with 99% query cover and 40.05% aa identity. Other top hits included crohivirus A (NC_025474) with 38.14% aa identity, and bovine parechovirus (LC790729) with 35.68% aa identity, both belonging to the family *Picornaviridae*.

**Fig. 6:**
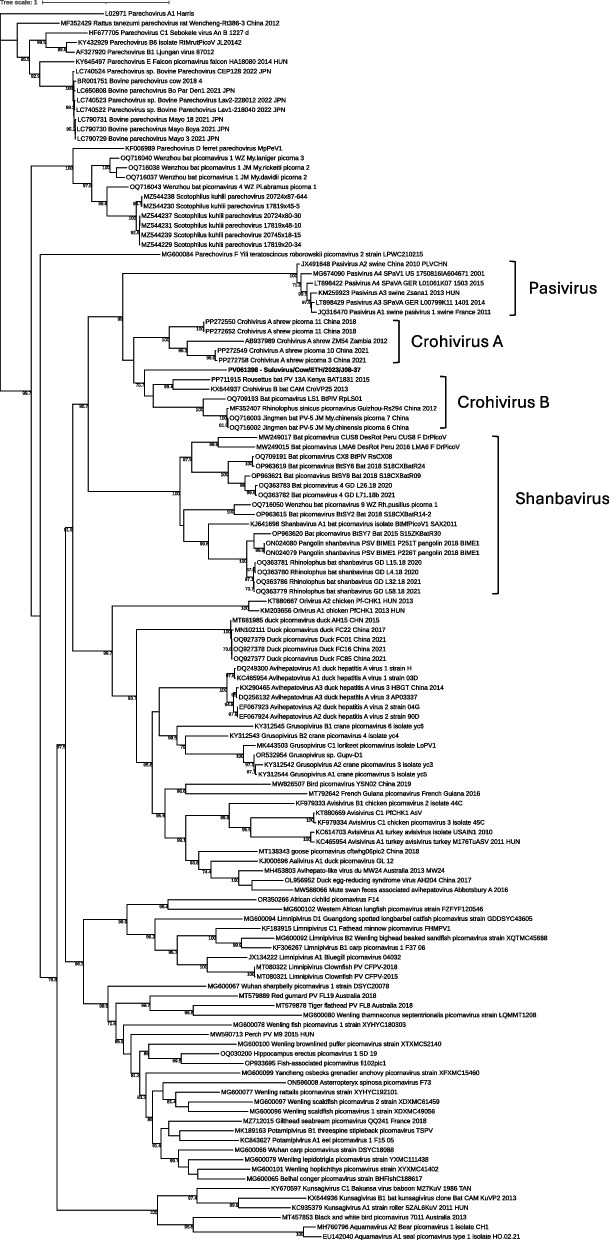
Maximum likelihood tree of the 3D region aa sequence of the *Paaviviridae* subfamily with bootstrap values ≥ 70 displayed. Suluvirus is indicated by bold text (PV061398)

**Fig. 7:**
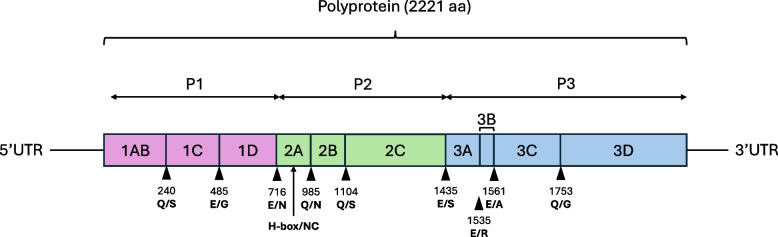
Schematic figure of the suluvirus polyprotein (2221 aa) showing the proposed cleavage sites and motifs

Phylogenetic analysis of the 3D and P1 regions showed that suluvirus clusters with crohiviruses in the 3D region (Fig. 6) and forms its separate branch in the P1 region (Supplementary Fig. 1). Analysis of the polyprotein sequence revealed that suluvirus has the genome constellation 5′-UTR-1AB-1C-1D/2A^H−box/NC^−2B-2C/3A-3B-3C-3D]−3′-UTR, similar to members of the crohivirus genus. However, suluvirus lacks the NPGP-motif in 2A in contrast to its closest phylogenetic relatives, crohivirus and pasivirus (Fig. 7). Further details on alternative cleavage sites and motifs can be found in Supplementary File 1. With a similar genome structure and protein composition like other viruses in the *Picornaviridae* family, in combination with the BLAST and phylogenetic results, we propose that suluvirus is either a new genus within the *Picornaviridae* family or a new species within the crohivirus genus.

#### Screening of individual viruses

The investigated samples have in a previously published study been screened, using qPCR, for RVA and BCoV [13]. In the present study, qPCR was used to also screen the samples for BNoV and suluvirus. When combining the results for the four viruses it was observed that all viruses could be detected in both diarrheic and non-diarrheic calves (Table 3). In total, 20/47 calves tested positive for at least one of the four viruses, however, only two calves tested positive for more than one virus. In the two samples with a co-infection, RVA and suluvirus, as well as BCoV and suluvirus, respectively, were identified. Additional details on number of positive samples in each sequencing pool is available in Supplementary Table 3.

**Table 3:** Detection of RVA, BCoV, BNoV and suluvirus in fecal samples from diarrheic and non-diarrheic animals. Data on RVA and BCoV were previously reported by Bergholm *et. al.* [13], while the results for BNoV and suluvirus are newly presented here for the first time

Virus	Diarrheic	Non-Diarrheic	Total
RVA	3/26	6/21	9/47
BCoV	3/26	1/21	4/47
BNoV	2/26	1/21	3/47
Suluvirus	2/26	4/21	6/47

## Discussion

Our viral metagenomic analysis revealed a large number of RNA, as well as DNA viruses, in both diarrheic and non-diarrheic calves from central Ethiopia. As seen in Fig. 1A, one of the most abundant viral families across the sequencing pools was *Caulimoviridae,* a family of DNA viruses that infect plants. Another virus family that infects plants, *Tombusviridae*, was also among the most abundant. Furthermore, viral families that infect prokaryotes were also found in all sequencing pools; *Suoliviridae, Steitzviridae,* and *Peduoviridae.* The presence of plant and prokaryotic viruses in the virome is not surprising, since plants are a part of the diet and as a large number of bacteria is present in fecal material. Furthermore, the viral families *Parvoviridae, Tobaniviridae, Coronaviridae, Sedoreoviridae,* and *Picornaviridae* were among the most abundant on read level and include viruses that have been linked to enteric disease in cattle. To further investigate the abundance of mammalian viruses, we removed reads assigned to viral families that infect plants, fungi, archaea, and invertebrates (Fig. 1B). We see a similar pattern of viral families in both Fig. 1A and B, with the enteric viruses *Parvoviridae, Tobaniviridae, Coronaviridae, Sedoreoviridae,* and *Picornaviridae* being among the most abundant. Among the mammalian viruses we also see *Astroviridae*, a viral family associated with enteric disease*,* as well as *Circoviridae, Orthoherpesviridae*, *Poxviridae*, and *Retroviridae* (Fig. 1B). However, the last four are not mainly associated with enteric disease. Of the enteric viral families, reads classified as *Parvoviridae* and *Picornaviridae* could be found in all sequencing pools (Fig. 1A and B). Viruses within the *Picornaviridae family* were abundant in both the diarrheic and non-diarrheic pools, indicating that viruses within this viral family could be a part of the normal viral flora. This could mean that other viruses of lower abundance or pathogens of non-viral origin are causing, or contributing to, disease in the diarrheic pools that have similar virome composition as the non-diarrheic pools. Interestingly four pools deviate in their viral composition. In pool J02, a majority of the viral reads belonged to the *Tobaniviridae* family, in pool J04 the majority of the reads were classified as *Sedoreoviridae*, and in pool J05 and J07 the most abundant family was *Coronaviridae*. Viruses within *Coronaviridae* are known to cause diarrheic disease in cattle, and the large abundance of this viral family in two diarrheic pools (J05 and J07) further supports this. Viruses within the *Sedoreoviridae* family are also causative agents in calf diarrhea, however, here we see a large abundance of viral reads classified as *Sedoreoviridae* in a non-diarrheic pool (J04). Three calves in pool J04 tested positive for RVA by qPCR (Supplementary Table 3), with one having a low ct value (< 20; data now shown) [13]. This could indicate that the calf was sampled early in infection when the viral load is high, but before the onset of symptoms, giving a possible explanation for the large abundance of RVA reads in the non-diarrheic sequencing pool. The same explanation could be possible for the large abundance of *Tobaniviridae* reads in the non-diarrheic pool J02. In summary, we see a large diversity of viral families in the sequencing pools, with many of them associated with enteric disease.

Investigation of longer viral contigs identified sequences belonging to RVA within the *Sedoreoviridae* family. Several genotypes of RVA circulate in cattle, with the most prevalent genotypes being G6, G8, and G10 together with P[1], P[5], and P[11]. However, more unusual RVA genotypes have been identified in cattle on multiple occasions [28]. From pool J02, nine RVA segments could be characterized, revealing the genotype to be G24P[33], with the full genome constellation being G24-P[33]-I2-R2-C2-M2-Ax-N2-T9-E2-Hx. This correlates with the finding from our previous study, where the same sample was characterized as having the G24 G-type, but any further genotyping using conventional PCR was unsuccessful [13]. The G24P[33] genotype has been detected only twice prior to this study, in two cows in Japan (Dai-10), and once in cattle in Uruguay [29, 30]. The strain identified in this study, RVA/Cow/ETH/02/2023/G24P[33], shares the same genome constellation as Dai-10 from Japan, except for the NSP1 and NSP5 segments that remain unknown. Interestingly, four segments, VP1, VP2, VP3, and VP6 had the highest nucleotide identity with RVA strains isolated from humans (Table 2). The G24P[33] genotype is hypothesized to have arisen through interspecies transmission and reassortment, with multiple segments having a non-bovine origin. In addition, a G24P[14] genotype has also been isolated from a human in the USA [31], and the T9 genotype (NSP3) has been associated with other atypical RVA genotypes [29, 32]. The G24P[33] genotype has now been identified on three separate continents, indicating that even if being unusual, it is circulating in the cattle population.

BCoV is associated with enteric disease in cattle and is also known to cross the species barrier, primarily to other ruminant species [33]. Furthermore, BCoV strains are believed to cluster according to geographical spread in correlation to international cattle trade, having established two separate lineages: the American/Asian lineage (US wild ruminant) and the European (Classical) lineage [34, 35]. In this study, we identified two complete genome sequences of BCoV from two separate sequencing pools. Phylogenetic analysis of the genomes showed that BCoV/Cow/ETH/2023/25 cluster together with BCoV strains from the European (Classical) lineage, while BCoV/Cow/ETH/2023/40 group with viruses isolated from dromedary camels, forming a distinct clade of DcCoVs separate from both the American/Asian and European lineage (Fig. 2). This shows that two distinct strains of BCoVs are present in the Ethiopian calves, both a classical BCoV strain and a strain more similar to DcCoVs. BCoV/Cow/ETH/2023/40 was most similar to a DcCoV isolated from a dromedary camel in Ethiopia [36], suggesting that the infected calf has either been directly exposed through contact with dromedary camels, or that a DcCoV has previously been introduced into the cattle population and is now circulating together with classical BCoV strains. This supports the findings from our previous study, where we analyzed the partial spike sequence of BCoV-positive samples that are included in the present sequencing pools [13]. This provides further evidence of the ability of BCoV to cross the species barrier and establishing new strains in both domestic and wild animals.

In addition to the well-known enteric pathogens RVA and BCoV multiple RNA viruses associated to diarrheic disease in calves were discovered through the metagenomic analysis. This included the viruses, BoAstV, BNoV, nebovirus, BKV, BEV, HuV, BooV, and BToV, which were all genetically characterized for the first time in cattle in Ethiopia. However, no complete genomes belonging to enteric DNA viruses were found, most likely due to the sequencing material being RNA and that most of the remaining DNA was degraded as part of the sequencing preparation.

BoAstV has been linked to both enteric and neurotropic disease in cattle [37] and here we identified three BoAstV genotypes. BoAstV/Cow/ETH/2023/J04 was genotyped as group 5, BoAstV/Cow/ETH/2023/J08 as group 2, and BoAstV/Cow/ETH/2023/J09 as group 4. BoAstV sequences within group 4 and 5 are derived from diarrheic and non-diarrheic animals and are believed to be associated with the enteric version of the virus. Group 2 contains sequences identified in cattle with enteric, neurotropic, and respiratory symptoms [26, 37]. This shows that at least three genotypes of BoAstV are present within the Ethiopian cattle population. In pool J04 (Non-diarrheic, Holeta Farm Y), we also found a complete sequence of BNoV GIII. Based on phylogeny it was further characterized as belonging to genotype GIII.2, which is the most common of the four known genotypes in cattle. Furthermore, a partial genome sequence of nebovirus was detected in a diarrheic sequencing pool (J09). Both BoAstV, BNoV, and neboviruses have been detected in diarrheic and non-diarrheic animals worldwide, including in Egypt and Tunisia [38, 39]. However, to our knowledge, this is the first detection in Ethiopia and in the Sub-Saharan region.

*Picornaviridae* is a large and diverse family of viruses, and it includes several viruses linked to diarrheic disease in both cattle and other animals. BKV was first detected in 2003 and has since then been detected in many parts of the world, both in diarrheic and healthy animals. However, BKVs role in calf diarrhea remains unclear due to its presence in both diarrheic and healthy animals [40]. Our study shows similar results, with BKV sequences identified in all sequencing pools (both diarrheic and non-diarrheic). BEV consist of two subgroups (E and F) and has been detected in cattle suffering from enteric disease, however, the infection is not believed to be a major contributor to calf diarrhea [2]. We found two full-length BEV genomes in two of the diarrheic sequencing pools (J01 and J03) and genotyping of the sequences revealed two genotypes, F2 and F7, within the subgroup BEV-F. The F2 genotype has been reported in several countries, including Egypt [41], while F7 is a new genotype, discovered in Japan in 2017 [42]. Our findings demonstrate that at least two genotypes of BEV are present within the Ethiopian cattle population. HuV and BooV are fairly new viruses within the *Picornaviridae* family, detected in cattle in Hungary in 2008 (HuV) and in Japan in 2009 (BooV) [9, 43]. In the present study, HuV was identified in both diarrheic and non-diarrheic animals, indicating that it might not be the primary cause of diarrheic disease, at least not as the sole agent. This correlates with the original study from Hungary, where HuV was found in non-diarrheic cattle. However, with few studies available on HuV in cattle, and this being the first detection in Africa, more data is needed to understand the potential role of HuV in calf diarrhea. A complete BooV genome sequence was found in a non-diarrheic pool (J04) and was most similar to BooV B strains on both nt and aa level. Currently, there are three known species of BooV: A, B and C, with A and B known to infect cattle. Of the data available, BooV B seems to be the most common strain in cattle [44, 45]. However, there is still only a few studies available investigating BooV in cattle, and with this study being the first in Africa more research on BooV is needed. Lastly, BToV, was identified in pool J02 (Non-diarrheic, Sebeta). It was most similar to BToVs from China, however, with a lack of full-length BToV sequences from other countries it is difficult to draw any further conclusions on phylogeny and relationship with other BToV isolates.

In addition to the known enteric RNA viruses, we identified a putative novel virus, suluvirus, within the *Picornaviridae* family. *Picornaviridae* is large and diverse family, consisting of 63 genera and over 147 species and several unassigned viruses [46]. Many viruses within the family have been detected in recent years, including viruses that infect cattle [9, 43]. Using DIAMOND, suluvirus was classified within the *Pisoniviricetes* class, with the closest match being crohivirus. Investigation of the polyprotein sequence and genome layout together with phylogenetic analysis supported the conclusion that suluvirus is a new virus within the *Picornaviridae* family. Whether suluvirus is a new genus or a new species together with crohivirus A and B, is still uncertain. However, crohivirus A has so far only been detected in shrews, and crohivirus B only in bats [47, 48]. Providing further support that suluvirus, identified in a bovine host, would be a distinct species within crohiviruses, if not its own genus. Furthermore, qPCR screening revealed suluvirus in 6/47 calves from four different farms, indicating that suluvirus is a bovine enteric virus and that the detection using metagenomics was not an isolated occurrence. However, further investigation is needed to determine whether suluvirus has any role in calf diarrhea.

When screening individual samples for selected viruses identified in the metagenomic analysis, RVA, BCoV, BNoV, and suluvirus could all be detected in both diarrheic and non-diarrheic calves. Due to the small sample size no clear conclusion could be drawn on the association of the selected viruses with diarrhea. However, future studies including a larger sample size from a wider geographical area could provide a better understanding of the prevalence and role of the detected viruses in calf diarrhea in Ethiopia, especially in the context of co-infections. Furthermore, by including the sequencing of DNA viruses a complete picture of the virome could be achieved, enabling further characterization of the *Parvoviridae* family and other possible enteric DNA viruses.

## Conclusions

To conclude, this study is the first viral metagenomic study on calves in Ethiopia and it increases the knowledge on the enteric RNA viruses that circulate in the Ethiopian cattle population. The number of viruses is vast and the virome complex, and no clearcut picture can be seen in most cases of one virus being singly causative agent of calf diarrhea at this point. Furthermore, we discovered a putative novel virus, suluvirus. Continued research using viral metagenomics in both cattle and other livestock animals could provide further insights and better preparedness for both animal and human diseases.

## Supplementary Information

Supplementary Material 1.

Supplementary Material 2.

Supplementary Material 3.

## Data Availability

The raw sequence reads generated in this study are available at the NCBI sequence read archive (SRA) database under Bioproject accession (PRJNA1224038). All viral genome sequences have been deposited to GenBank under the accession numbers PV053516, PV061389-PV061398, and PV076094-PV076105.
