# Supplementary figures and images for: Metagenomic insights into the complex viral composition of the enteric RNA virome in healthy and diarrheic calves from Ethiopia
Source: Virol J. 2025 Jun 7;22:188. doi: 10.1186/s12985-025-02821-8 (PMC12145588; doi:10.1186/s12985-025-02821-8)

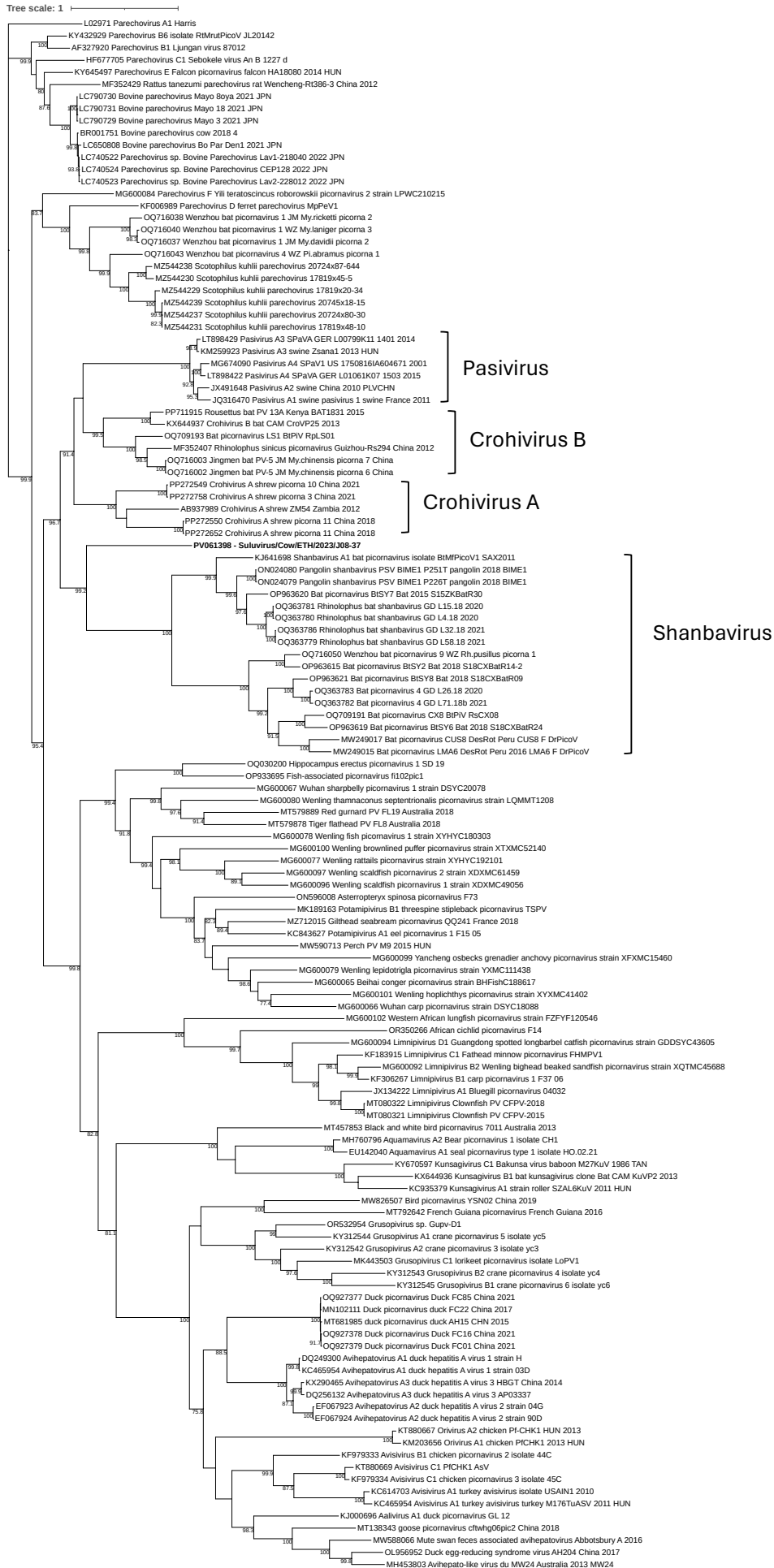

Supplement: Supplementary file 1 — Supplementary Material 1. [file 12985_2025_2821_MOESM1_ESM.pdf]
