# Supplementary material for: Metagenomic insights into the complex viral composition of the enteric RNA virome in healthy and diarrheic calves from Ethiopia
Source: Virol J. 2025 Jun 7;22:188. doi: 10.1186/s12985-025-02821-8 (PMC12145588; doi:10.1186/s12985-025-02821-8)
Supplement: Supplementary file 2 — Supplementary Material 2. [file 12985_2025_2821_MOESM2_ESM.pdf]

Suluvirus_polyprotein

Possible alternative cleavages

Motif

1AB

MEKISNIVDTLLNDPVTEEKEQTSDRISGVTTANASSIIQAAVKPLIPRNSYLGSKDDFYSCDYDP
MTAERNVRKMVQLGVYDWNGSATFMQEVFTIGLPKAFFPVDNPGSGFAHFFKYMRGSMHFQ
VQINAVTGAAGGLLVVYVPVGVDFSKICYASILNLPNCIVNIGTTSCIQMTVPYTNNKNFSLIRSTE
MGHLKGLVWAQYQCSSGSPTNCEVTVLGSLEQAQF**QG**PIPYTTVSQ

1C

SPSKWNKIKYSRNSVNICEGVGAMNLANSRWTSSAPSSALRGERMNVDYHTSGSDQPVRDF
VEIAQIPSLIRLPNVSFDNSGDSTRISRFNWQASTTKNTKISEFDLAPFQIANIFKLSSGFSCYRGS
IVLSFTCFNSPFHKGRFKVVINPCNAGSISTVGQTNALLYTICDIGLNSTFDITIPYTSQCWCSPLD
VSSAWRVEVWCINRLQYNPSCKSAVDFVVTARGGKDFQLLYPRDSDTRWE

1D

GLQSWGSEMDLIDPLDSDDEEQHVNVEQGKSDIASNLGLASVENTGDNTGPLPQQVALNYR
LHKPKLPRASHSRVDFFFGRAWWAGAIRPSAVTGQQRLDKPRTGRASLMDFFTYWSGELNIH
VINETSVYTEVTHDYINDLSSASNISGYGSWLVPPKQACTVNRPFYSDVPLRILESDGCFGFVRV
NNYENTNISMPFFISLRDSNFFRIKPFNK**QG**TWRSVVRSLDQE

2A

NLSPAQLLRCIEHIES**DEYD**ENAIFQMPVVIEHRTGLEKAKALSRYYTESALRYFSKVKNSDEME
SEGLTDAQYYKLYYNLSSFEKTLLADTLLYSINGKYMMSKYQGTRVSYKFILCYMALHSGKSLNQ
LNSQNINLELVYKHRALYR**HYGI**RMHGKIIHLNTQNVLDIFNNVNCEVVCEDDDGNWIVEQVI
PASSFMCDKLINFIGTRHKFSCNF**NC**ETFARSFFPESEISQSKSLFLFGSILVVCTMSHFLVGDTT
SQSIDEICSQ

2B

NNDGIFSRAMSWFSNAFVDVTHSKLISIFVKSIIRLVCYLIMYCHAPNVPTSLALATLIFMDVQAA
GVACDESKGLVKCLLHGDIKGLVSGILEKIQMVDEDKSELMKDTMDSLSMFSQ

2C

SPFDEYNKGSLAAKNTLWWIDLLKDIATKLKRIFKPTQSQIFQKWLSENEDLVANLLATVNEHLK
SCKSATFLRIPDNVTKHRFLVSKMIQLSLAFSRFSPTSSFSAQVKHLKDALVNVVIPKPVNEQVTR
IEPLGICIH**GASGQGKS**FLSHMIIRDLKKNNKWNSDDVYTHAVGSQYYDGYHEQKI**HVIDD**MG
QNKDELDIKVLCQAISSVPFIVPMASVEEKGTLYKSEVVIATTNKFDFSTFTLIDQEALKRRFPIDVF
IRARKEFTRNGKLDVHNYMDRVKEGQVWEVSVDGYKWENLDYQKFINDVQMQLNSRKNSFK
IWTEFINE

3A

SPEDEKEIDFNDWSVQFLNDFKDPKVVFDWDIVENKSSVLVKSNSTIKNWVKRKINDLKDFITK
NQGWFTICSLVASGVGLLATVVYFVNKHKEKPIKEE

3B

RA**Y**NPSIQVKADKRLMLGKTINLSSE

3C

APYLSEMSHLLEQCAYINDGKTTIHCFPLHSNKMVVYNHSRFILNLMDKPSLFYKNKQFEITSW
QFQTLLYKNEPMDLAIITIDNLPFRFKNNVKFISSVYGSDSMLIWNSPHGPLAMPVDSVSDHGY
NITRQGDVCAQSISYNCQTAA**GMCGG**LLISRVDGAFKIHGMHIAGNGVVGRSASLLCLKSFES
Q

3D

GLILAKTSTPLYVHQPAKTKLKESILHGIWPVQMQPAVLSQNDDRLMVPVESMIKHSACKYNVN
VFEPNLNVFNYIKIELKKVFFDLFGKNDVMSLQEAVLCPDEHKLDLTTSPGLKYTNRGLRKSDLV
DRDKKWISPVLICDVNKLNEDLVSGKQMDVKFYAHL**KDELR**DISKIADGNTRCIEASDFDYVVL
HRIYYGNLYKKIYDTPAYLSGLAVGINPWCDWHTMINSLYEYCYDLDFKKFDGSLSKELMGHAA
DVISYCLENPIIGRRLLDPVIESKHIVLDEMWEVY**GGMPSG**SPCTTVLNSICNLLVCGTLCYVTTP
GKFKMIV**YGDD**VIISTDSPLDTDKFVELAKEWFGMVVTSGDKKNKILDKRPYDVV**FLKR**ICKNFP
GSSTYVGALQIDTIEQHIMWCKNKRTFENQLYSALCEMFLHGEDEYNNFVKKIEKRCRKFGINV
RPYDLIGSDMVQFVYG
