# Supplementary material for: Metagenomic insights into the complex viral composition of the enteric RNA virome in healthy and diarrheic calves from Ethiopia
Source: Virol J. 2025 Jun 7;22:188. doi: 10.1186/s12985-025-02821-8 (PMC12145588; doi:10.1186/s12985-025-02821-8)
Supplement: Supplementary file 3 — Supplementary Material 3. [file 12985_2025_2821_MOESM3_ESM.pdf]

Supplementary Table 1. List of viral sequences and their GenBank accession numbers.

**Viral sequence**	**GenBank accession**
BNoV/Cow/ETH/2023/J04	PV053516
BToV/Cow/ETH/2023/J02	PV061389
BCoV/Cow/ETH/2023/25	PV061390
BCoV/Cow/ETH/2023/40	PV061391
HuV/Cow/ETH/2023/J07	PV061392
BooV/Cow/ETH/2023/J06	PV061393
BKV/Cow/ETH/2023/J04	PV061394
EV-F2/Cow/ETH/2023/J01	PV061395
EV-F7/Cow/ETH/2023/J03	PV061396
Nebovirus/Cow/ETH/2023/J09	PV061397
Suluvirus/Cow/ETH/2023/J08-37	PV061398
BoAstV/Cow/ETH/2023/J04	PV076094
BoAstV/Cow/ETH/2023/J09	PV076095
BoAstV/Cow/ETH/2023/J08	PV076096
RVA/Cow/ETH/02/2023/G24P[33] (VP7)	PV076097
RVA/Cow/ETH/02/2023/G24P[33] (NSP2)	PV076098
RVA/Cow/ETH/02/2023/G24P[33] (VP2)	PV076099
RVA/Cow/ETH/02/2023/G24P[33] (NSP3)	PV076100
RVA/Cow/ETH/02/2023/G24P[33] (NSP4)	PV076101
RVA/Cow/ETH/02/2023/G24P[33] (VP6)	PV076102
RVA/Cow/ETH/02/2023/G24P[33] (VP4)	PV076103
RVA/Cow/ETH/02/2023/G24P[33] (VP1)	PV076104
RVA/Cow/ETH/02/2023/G24P[33] (VP3)	PV076105

Supplementary Table 2. List of primers/probes used in the screening of individual viruses.

Virus	Primer/probe sequence (5’-3’)	Reference
BNoV	Fw: TCCATGTTYGCHTGGATG<br>Rv: TCAGTCATCTTCATTTACAAAATC<br>Pb: FAM-TGTGGGAAGGTAGTCGCGACRYC–BHQ1	1.
Suluvirus	Fw: TGGCAGTGGGCATAAATCCAT<br>Rv: TCAGCAGCATGTCCCATCAA	This study
1. Development of real-time RT-PCR for detection of bovine nebovirus and norovirus in fecal samples. Unpublished manuscript.		

Supplementary Table 3. Number of virus-positive samples for each sequencing pool.

Virus	J01 (D)	J02 (H)	J03 (D)	J04 (H)	J05 (D)	J06 (H)	J07 (D)	J08 (H)	J09 (D)	J10 (H)	Total (D)	Total (H)
RVA	0/7	1/5	1/6	3/6	2/5	2/4	0/6	0/4	0/2	0/2	3/26	6/21
BCoV	0/7	0/5	0/6	0/6	2/5	0/4	1/6	1/4	0/2	0/2	3/26	1/21
BNoV	0/7	0/5	1/6	1/6	0/5	0/4	1/6	0/4	0/2	0/2	2/26	1/21
Suluvirus	2/7	1/5	0/6	0/6	0/5	0/4	0/6	3/4	0/2	0/2	2/26	4/21
